# 

**Published:** 1914-07-25

**Authors:** 


### CONTENTS.

PAGE

**Certain Provincial Hospitals: Their Dissolution or Reform** ... ... ... ... ... 453

**Hospitals and the Dust Nuisance** ... ... 454

**Hospital and Institutional News** ... ... ... 455

A Novel Legal Decision—Action Against the Blackpool Board of Management—The Blackpool Case: Judgment—Employers' Subscriptions and Compensation Cases—Important Gift to King Edward VII. Hospital, Cardiff—Growth of the Pay-ward Movement—The East Sussex Hospital Foundation-stone Laid—The Medical Deputation and Forcible Feeding—The Employment of Consumptives—The Medical Inspection of Merchant Ships—Hospital Investments—Board's Gift to a Hospital Secretary—The Winsley Sanatorium Difficulties—Cinematography at a Hospital Opening—A Chest Hospital Transferred to Public Control—The Last Report of the Lunacy Commissioners—Mental Wards in Workhouses—The "Parade Season"—Hospital Officers at Play—The Question of Special Departments for Venereal Diseases—The Power of a Linen League—Further Bequests from Sir Alfred Jones—Scotch Servants and Hospital Problems—The New Workhouse Hospital at Hull—The Visitation of Public Institutions—A Hospital Equerry for the King—This Week's Drug Market.

**Medico-Psychology** ... ... ... ... ... 461

The Problem of Functional Paralysis. [*See over.*]

### CONTENTS—(*continued*).

PAGE

**Research and Remedies** ... ... ... ... ... 462
Collargol Pyelography: A Warning—The Treatment of Ankylostoma Anæmia.

**The Portrait of a Great Founder** ... ... 463

The History of Charing Cross Hospital and School.

**The Administration of the Willesden Workhouse** ... ... ... ... ... ... ... ... 464

**Reports on Hospitals of the United Kingdom**— 465
By SIR HENRY BURDETT, K.C.B., K.C.V.O.
The Royal Southern Hospital, Liverpool.
The Holmesdale Cottage Hospital, Sevenoaks.

**The Ambulance Attendant: A Curious Case...** 466

**National Insurance Act Developments** ... ... 467
Disablement Benefit—the Last of the Benefits to Come into Operation.

**Panel Practitioners and Chemists** ... ... 468

**The Progress of Mental Treatment in Scotland** 469
Boarding-out or Institutional Treatment?

**Economy in the Generation and Use of Steam** 470

III.—Prevention of Waste.

**Working Men and Representation** ... ... 471
Mr. Roden Orde on the Principle and its Application.

**The Editor's Letter-Box** ... ... ... ... ... 473
The Metropolitan Hospital—The Royal National Sanatorium.

**British Pharmaceutical Conference** ... ... 474

**Round the World** ... ... ... ... ... ... 475

Fellow-Workers and their Doings.

**The Department of Modern Nursing** ... ... 476

St. Mary's Hospital.

**The Profession of Medicine** ... ... ... 478
The Examination of Proposers for Life Insurance.
The Repetition of Prescriptions.

**The Institutional Worker** .. (Suppl.) 1
Points in Institutional Housekeeping.
Another Hospital Worthy.

